# P02.58. The feasibility of integrating ear acupuncture into the aeromedical evacuation system from Ramstein Air Base (Germany) to Andrews Air Force Base (US)

**DOI:** 10.1186/1472-6882-12-S1-P114

**Published:** 2012-06-12

**Authors:** J Walter, A York, S Thati, R Niemtzow, S Burns

**Affiliations:** 1 Samueli Institute, Alexandria, USA; 2 U.S. Air Force, Andrews AFB, USA

## Purpose

Recently, military leaders have endorsed the need for holistic, non-pharmacologically based approaches to pain. Acupuncture has been shown to be an effective adjunctive therapy for specific pain conditions; however, acupuncture has occupied only a marginal role in pain management. We investigated what would happen if a simple ear acupuncture procedure was introduced into the medical care delivered in the aeromedical evacuation system to wounded warfighters being evacuated from Germany to the US.

## Methods

Volunteers were recruited among military personnel being evacuated from Germany to the U.S. who reported pain. Five semi-permanent needles were placed in each ear just prior to flight. Pain scores were recorded at intervals during and at the end of the flight. Patient satisfaction scores and medication use were measured. Medical personnel provided information on provider satisfaction. Needles were removed upon landing.

## Results

With advance preparation and training of personnel, the administration of an ear acupuncture procedure did not interfere with the normal pre-flight preparation process. A larger, randomized controlled clinical trial aimed at assessing effectiveness of ear acupuncture is possible. Fifty percent reported being mostly satisfied and 21% were very satisfied with the ear acupuncture treatment. Sixty-two percent reported they would elect to receive the ear acupuncture treatment again for pain relief. There was a significant decrease (p<0.0001) in pain levels (avg. pre-treatment pain rate=4.07; post-flight pain rate=2.76). No significant difference was detected between participants who reported taking pain medications and those who did not therefore both groups (medication takers and non-takers) experienced similar decreases in pain levels.

## Conclusion

The potential of acupuncture to deliver pain relief with minimal side effects in this population is worth further investigation, especially if it may mitigate the overuse of pain medication with its inherent risks and challenges. We will discuss the challenges of conducting research in this environment and the details of the results.

